# Relationship between Bone Mineral Density and Serum Osteoprotegerin in Patients with Chronic Heart Failure

**DOI:** 10.1371/journal.pone.0044242

**Published:** 2012-08-30

**Authors:** Ying-Hsien Chen, Yen-Wen Wu, Wei-Shiung Yang, Shoei-Shen Wang, Chi-Ming Lee, Nai-Kuan Chou, Ron-Bin Hsu, Yen-Hung Lin, Mao-Shin Lin, Yi-Lwun Ho, Ming-Fong Chen

**Affiliations:** 1 National Taiwan University College of Medicine, Taipei, Taiwan; 2 Department of Internal Medicine, National Taiwan University Hospital, Taipei, Taiwan; 3 Department of Nuclear Medicine, National Taiwan University Hospital, Taipei, Taiwan; 4 Department of Surgery, National Taiwan University Hospital, Taipei, Taiwan; 5 Cardiovascular Center, National Taiwan University Hospital Yun-Lin Branch, Dou-Liu City, Taiwan; 6 Department of Nuclear Medicine, Far Eastern Memorial Hospital, New Taipei City, Taiwan; UAE University, Faculty of Medicine & Health Sciences, United Arab Emirates

## Abstract

**Purpose:**

Heart failure (HF) had been reported with increased risk of hip fractures. However, the relationship between circulating biomarkers and bone mineral density (BMD) in chronic HF remained unclear.

**Methods:**

This is a cross-sectional study which recruited stable chronic HF from registry of the Heart Failure Center of National Taiwan University Hospital. Patients underwent dual-energy x-ray absorptiometry (DEXA) measurements at hip and lumbar spines and biochemical assessments including B-type natriuretic peptide (BNP-32), myostatin, follistatin and osteoprotegerin (OPG).

**Results:**

A total of 115 stable chronic HF individuals with left ventricular ejection fraction (EF) <45% (74% of male, mean age at 59) were recruited with 24 patients in NYHA class I, 73 patients in NYHA class II and 18 patients in NYHA class III. Results of BMD showed that Z scores of hip in NYHA III group (−0.12±1.15) was significantly lower than who were NYHA II (0.58±1.04). Serum OPG was significantly higher in subjects of NYHA III (9.3±4.6 pmol/l) than NYHA II (7.4±2.8 pmol/l) or NYHA I (6.8±3.6 pmol/l) groups. There’s a significant negative association between log transformed serum OPG and trochanteric BMD (R = −0.299, P = 0.001), which remained significant after multivariate analysis.

**Conclusions:**

Our study demonstrated an inverse association between serum OPG and trochanteric BMD in patients with HF. OPG may be a predictor of BMD and an alternative to DEXA for identifying at risk HF patients for osteoporosis.

## Introduction

Heart failure (HF) and osteoporosis, both as disabling conditions, are two common chronic conditions in elderly which are gaining importance for healthcare recently due to the associated significant morbidity and mortality [1]. These two disabling conditions adversely affect quality of life especially in frail elderly individuals. From recent large epidemiological study, HF is associated with a substantial increase in osteoporotic fractures, particularly in the hip region [2]. In addition to decreased physical performance in HF and sharing a number of common risk factors [3] such as older age, smoking, renal insufficiency and type 2 diabetes, accelerated bone loss may also come from altered vitamin D levels, hyperparathyroidism [4] elevated aldosterone levels [5], elevated fibrotic markers [6] and loop diuretics use in subjects with HF [7]. Cardiac cachexia-related biomarkers including adiponection, follistatin and myostatin had been investigated in muscle, fat, and bone metabolism in heart failure metabolism [8], however, the relationship between circulating biomarkers and bone mineral density (BMD) in chronic HF remained unclear. To the best of our knowledge, HF is a clinical syndrome characterized by prolonged activation of the neuroendocrine system ranging from sympathoadrenal system, natriuretic peptides, renin-angiotensin-aldosterone system (RAAS) and to updated markers of osteoprotegerin (OPG) [9]. The higher OPG level predicts poor prognosis in subjects with HF with higher all-cause mortality and hospitalization for worsening of HF [10]. OPG has also been reported to be associated with neuroendocrine activation in elderly males with systolic HF [11] which makes believe that there were interaction among HF and osteoporosis through systemic hormonal activation. However, a direct causal association between these circulating biomarkers and osteoporosis or risk of fractures has not been established in elderly patients with chronic HF, nor does these associations hold true in all HF patients. Therefore, the present study was aimed to evaluate the associations between osteoporosis and HF in elderly patients, and to assess the circulating biomarkers on this association.

## Methods

### Patients

The registry of the specialized clinic of the Heart Failure Center of National Taiwan University Hospital since Aug. 2008 formed the basis for this study. All patients attending the clinic for the evaluation of heart failure were asked to participate in the registry and provide written informed consent for data storage and evaluation. The diagnosis of heart failure was based on the criteria of symptoms compatible with the diagnosis in the presence of objective evidence of systolic dysfunction which defined by left ventricular ejection fraction (LVEF) below 45% from echocardiography, radionuclide angiography and/or cine angiography. A total of 115 subjects with stable chronic systolic dysfunction were eligible and agreed to participate between January and October 2009. The main exclusion criteria were age <18 or >90 years old, residence outside the Great Taipei region, history of any non-traumatic fracture, metallic prosthesis/fixation at hip or lumbar spines, renal insufficiency with creatinine >3.0 mg/dL, advanced non-cardiac diseases or malignancy with an expected life expectancy less than 1 year, connective tissue or musculoskeletal diseases, significant liver, thyroid, adrenal gland or pituitary diseases, inability to ambulate, or taking hormone replacement therapy or steroids, unwillingness to participate or provide blood samples.

### Ethics Statement

The study protocol was approved by the Institutional Review Board at National Taiwan university Hospital before the initiation of the study. Written form informed consent was obtained from all participating patients.

### Study Design

A physical examination was performed to assess HF severity and New York Heart Association (NYHA) class and patients were classified accordingly. All participants were interrogated for cause of HF, medication history, smoking, alcohol abuse, additional comorbidities and previous falls and or fractures. Biochemical data within 3 months and echocardiography within six months were recorded. A priori medication classes of interest included angiotensin-converting enzyme inhibitors, angiotensin receptor blockers, beta-blockers, calcium channel blockers, lipid lower agents (statins, fibrates), antiplatelet agents, long-acting nitrate preparations, diuretics, spironolactone and thiazide diuretics.

### Bone Mineral Density (BMD) Measurements

BMD measurements using Dual-Energy X-ray Absorptiometry (DEXA, Norland XR-26, USA) were performed according standard protocols by a single experienced operator. Hip with femoral neck, trochanter, Wards Triangle and lumbar spines over L2–L4 regions were measured and expressed as g/cm^2^. The coefficient of variation of BMD measurement at the hip and lumbar spine was <1%. The difference between an individual’s BMD and the mean BMD for a reference population are expressed in standard deviation (SD) units. Z- and T-scores were calculated where the Z-score is the S.D. of the individual’s BMD compared to the mean BMD score of a similar sex-, age-, weight- and height matched population and the T-score is the S.D. of the individual’s BMD compared with the mean BMD score in a young healthy population. The coefficient of variation for DEXA was less than 3% in our lab. [12]

### Circulating Biomarkers Assessments

Peripheral venous blood was obtained at 8:00 AM after overnight fasting and refraining from smoking. Serum samples were immediately deep frozen and kept at −70°C until assay. Testing was performed using commercially available kits for brain natriuretic peptide 32 (BNP-32) (Phoenix Pharmaceuticals, Burlingame, CA, USA), high sensitivity C-Reactive Protein (hsCRP) (Chemicon International, Temecula, CA, USA), myostatin (Immundiagnostik AG, Bensheim, Germany), follistatin and Osteoprotegerin (R and D Systems, Minneapolis, MN, USA) following the manufacturer’s instructions. The results were interpolated from the standard reference curve provided with each kit. The inter-assay and intra-assay coefficients of variation of all assays were ≤10%, and all laboratory work was undertaken by researchers who were blinded to the patient’s clinical details. [13]

### Statistical Analysis

Continuous variables are expressed as mean ± SD. One-way ANOVA was used to compare continuous data of three different NYHA functional classes. Categorical variables are compared by Chi-square analysis or Fisher’s exact tests and summarized by proportion in each category. Pearson’s correlation test was used to analyze the association between bone mineral density and other clinical determinants. Data of blood urea nitrogen, creatinine, fasting glucose, total cholesterol, triglyceride, follistatin, myostatin, high sensitivity C reactive protein, BNP-32, and osteoprotegerin were log-transformed due to non-normality which was tested by the Shapiro-Francia W’ test. Significant determinants in the Pearson’s correlation test (P<0.05) were then tested by a multivariate linear regression test with stepwise subset selection to identify independent factors predicting bone mineral density. All statistical analyses are completed using the STATA 11.0 and SPSS 17.0 software package. A two-sided P<0.05 was considered statistically significant.

## Results

A total of 115 HF patients (74% of male, mean age at 59) who lived in Great Taipei Region were recruited in the outpatient clinics and grouped according to NYHA functional class. The majority of participants were of NYHA class I and II at the time of clinical evaluation (24 in the state of NYHA function class I, 73 in class II, 18 in class III and none in class IV). The clinical characteristics of patients according to NYHA class are presented in Table 1. There was a tendency of lower LVEF in advanced NYHA class (P = 0.06). The demographic characteristics, comorbidities, biochemical lab data and cardiac medications were not significantly different between groups except significantly lower rate of beta blocker use in advanced NYHA class (83% in NYHA class I, 60% in class II, vs. 44% in class III, P = 0.03), (Table 1).

**Table 1:** Clinical parameters, laboratory data and cardiac medication in different NYHA functional classes.

	Total population	NYHA I	NYHA II	NYHA III	P value
	N = 115	N = 24	N = 73	N = 18	
**Clinical parameters**					
Gender, Male (n, %)	85, 74	21, 88	53,73	11, 61	0.14
Age, year	59.2a±14.7	56.8±12.9	59.5±14.9	61.0±16.4	0.62
Age > = 60, (n, %)	58, 50.4	10, 41.7	39, 53.4	9, 50.0	0.61
Height, cm	165.2±8.4	167.4±6.1	165.0±9.3	163.0±6.7	0.22
Body weight, Kg	69.4±13.7	70.7±10.5	69.9±13.3	65.6±18.1	0.43
BMI, Kg/m^2^	25.4±4.4	25.3±3.7	25.5±3.9	24.6±6.7	0.71
CAD (n, %)	55, 47	13, 54	32, 44	10, 56	0.53
DM (n, %)	34, 30	6, 25	22, 30	6, 33	0.83
Hypertension (n, %)	57, 50	14, 58	33, 45	10, 56	0.47
Af (n, %)	35, 30	8, 33	31, 43	4, 22	0.71
Smoking (n, %)	33, 29	7, 29	24, 33	2, 11	0.19
LVEF (n, %)	35.8±11.2	40.6±13.1	34.7±10.0	33.7±11.2	0.06
**Lab data**					
Blood urea nitrogen, mg/dL#	21.6±7.8	20.5±7.6	21.1±8.0	20.9±7.7	0.62
Creatinine, mg/dl#	1.2±0.4	1.3±0.4	1.2±0.3	1.3±0.6	0.47
Uric acid, mg/dL	6.8±1.9	6.1±1.4	7.0±1.8	6.7±2.5	0.13
Potassium, mmol/L	4.2±0.5	4.3±0.4	4.2±0.5	4.2±0.5	0.71
Fasting glucose, mg/dL#	111.2±42.2	109.4±33.7	110.6±42.7	116.8±52.6	0.91
Total cholesterol, mg/dL#	185.5±51.4	170.3±41.7	185.6±37.3	207.7±96.5	0.07
Triglyceride, mg/dL#	143.8±92.6	138.2±60.1	146.8±104.4	139.2±79.3	0.91
**Cardiac Medications, (n,%)**					
Diuretics	93, 80	19, 79	58, 79	16, 89	0.65
Spironolactone	47, 41	10, 42	27, 37	10, 56	0.36
Calcium channel blocker	9, 8	1, 4	5, 7	3, 17	0.29
Antiplatelet	62, 54	15, 63	35, 48	12, 67	0.24
Digoxin	56, 49	8, 33	37, 51	11, 61	0.18
ACEI	32, 28	6, 33	20, 27	4, 22	0.73
ARB	65, 57	15, 63	43, 59	3, 39	0.25
β-blockers	72, 63	20, 83	44, 60	8, 44	0.03
Statin	42, 37	11, 46	27, 37	4, 22	0.29
Fibrate	8, 7	1, 4	5, 7	2, 11	0.69
Anti-arrhythmics	28, 24	5, 21	20, 27	3, 17	0.58
Thiazolidinedione	4, 4	0, 0	4, 5	0, 0	0.31
**Heart failure related biomarker**					
Follistatin, pg/mL#	2109.4±973.2	1968.1±993.3	2135.6±973.3	2191.2±982.4	0.39
Myostatin, ng/mL#	7.2±4.0	8.3±4.4	7.2±3.9	5.6±3.1	0.10
hsCRP, ng/mL#	3.5±6.3	3.6±8.4	3.3±6.1	3.8±3.8	0.36
BNP-32, ng/mL#	1.95±0.89	1.99±0.74	1.94±0.98	1.87±0.71	0.74
Osteoprotegerin, pmol/L#	7.6±3.4	6.8±3.6	7.4±2.8	9.3±4.6* †	0.04
**Bone mineral density**					
Femoral neck BMD, g/cm2	0.82±0.18	0.80±0.13	0.84±0.19	0.76±0.17	0.15
Trochanteric BMD, g/cm2	0.68±0.14	0.68±0.12	0.70±0.13	0.60±0.16†	0.02
Ward triangle BMD, g/cm2	0.59±0.16	0.58±0.14	0.60±0.16	0.54±0.17	0.27
Hip Z score	0.37±1.05	0.12±0.86	0.58±1.04	−0.12±1.15†	0.017
Hip T score	−0.90±1.25	−1.13±1.05	−0.73±1.21	−1.29±1.53	0.14
L2 BMD, g/,m2	0.98±0.21	0.94±0.20	1.01±0.21	0.93±0.23	0.17
L3 BMD, g/cm2	1.03±0.24	1.02±0.23	1.06±0.22	0.94±0.28	0.16
L4 BMD, g/cm2	1.05±0.23	1.02±0.22	1.07±0.74	10.01±0.21	0.39
L2–4 BMD, g/cm2	1.03±0.22	0.99±0.21	1.04±0.22	0.98±0.21	0.33
L2–4 Z-Score	0.48±1.30	0.23±1.39	0.66±1.25	0.08±1.32	0.14
L2–4 T-Score	−0.02±1.65	−0.13±1.49	0.11±1.73	−0.43±1.50	0.43

Values given as n, percentage or mean±SD. BMI: body mass index, CAD: coronary artery disease, DM: diabetes mellitus, Af: atrial fibrillation, LVEF: left ventricle ejection fraction, ACEI: angiotensin-converting enzyme inhibitors, ARB: Angiotensin II receptor blockers, CRP: C-reactive protein, BNP-32: Brain natriuretic peptide 32, BMD: bone mineral density.

# Variables were not normally distributed, and were log transformed for statistical analysis.

* p<0.05 vs NYHA I,

† p<0.05 vs NYHA II.

**Figure 1:**
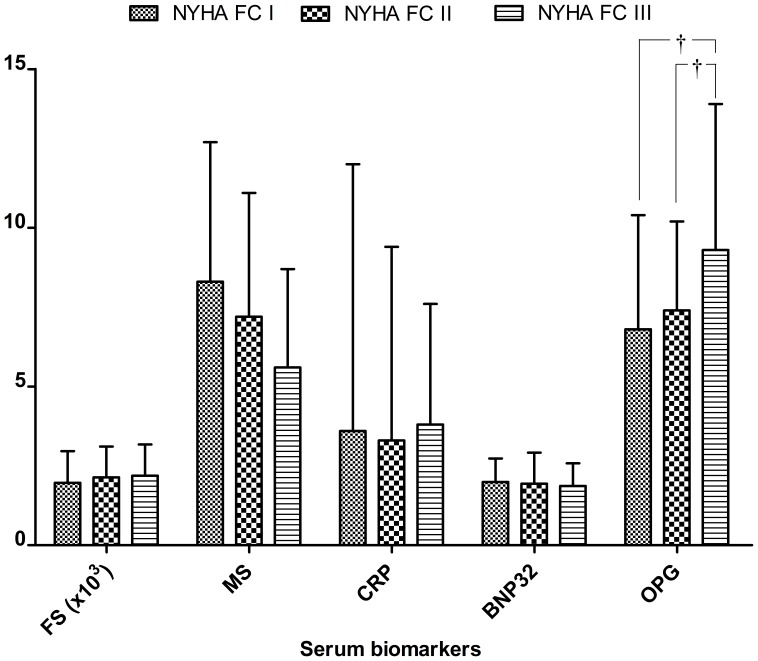
Heart failure related biomarker in different NYHA functional classes. FS: follistatin, MS: myostatin, BNP-32: Brain natriuretic peptide 32, CRP: high sensitivity C reactive protein, OPG: osteoprotegerin, †: p<0.05.

Of various circulating biomarkers (Table 1 and Figure 1), only OPG was associated with higher NYHA class (P = 0.04). There were no significant group differences in levels of follistatin, hsCRP or BNP-32. The lower myostatin levels were associated with higher NYHA class, but not reaching statistical significance (P = 0.10).

**Figure 2:**
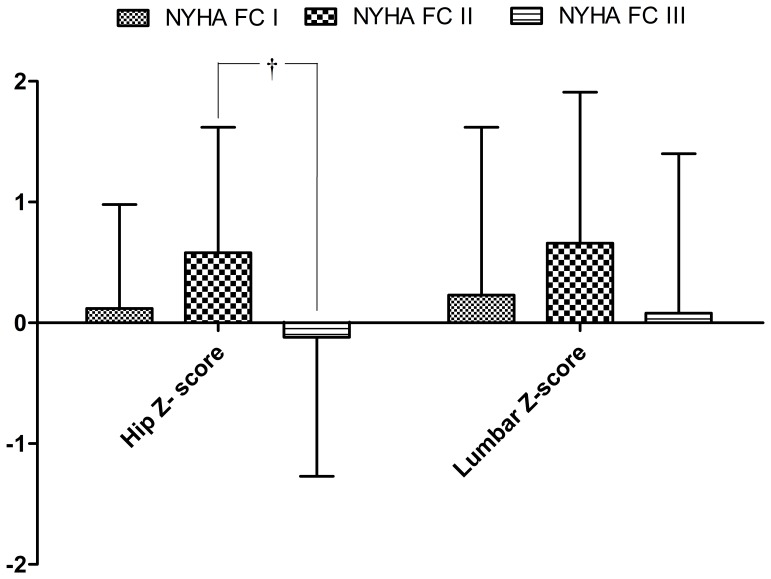
Hip and lumbar Z-score in different NYHA functional classes. A significant difference of hip Z-score was observed between NYHA functional class II and III, †: p<0.05.

BMD measurements of the hip and lumbar spines are shown in Table 1 and Figure 2. Comparisons of BMD of the femoral neck, trochanteric, Wards Triangle region of the hip and lumbar region revealed that a significant lower BMD over trochanteric region was observed in NYHA class III patients, with trochanteric BMD 0.60±0.16 g/cm^2^ comparing to NYHA class II 0.70±0.13 g/cm^2^ and NYHA class I 0.69±0.12 g/cm^2^ (P = 0.02). The hip Z-score in NYHA class III was –0.12±1.15, comparing to NYHA class II Z-score: 0.58±1.04 and NYHA class I Z score: 0.12±0.86 (P = 0.017). While in lumbar region, there is no significant difference in BMD among different NYHA functional classes.

Among all the HF related biomarkers, there was a significant inverse correlation between log transformed serum OPG and trochanteric BMD in patients with HF (R = −0.299, P = 0.001) (Table 2), femoral neck BMD (R = −0.29, P = 0.002) and Ward triangle region of hip (R = −0.34, P<0.001) while there is no significant correlation between OPG and lumbar region BMD. The correlation between clinical variables and trochanteric BMD was shown on Table 2. In additional to log transformed serum OPG, hypertension, age equal or more than 60, male gender, body weight, body height and body mass index was correlated with trochanteric BMD. The correlation between HF related biomarker and BMD was not observed in hsCRP, myostatin, follistatin or BNP-32.

**Table 2:** Factors associated with trochanteric bone mineral density.

	Pearson correlation coefficient	P value
Osteoprotegern#	−0.299	0.001
Male gender	0.420	<0.001
Age equal or more than 60	−0.2571	0.006
Hypertension	0.206	0.03
Smoking	0.181	0.05
Left ventricle systolic function	0.026	0.78
Diuretics	0.102	0.28
β-blockers	0.07	0.461
Blood urea nitrogen#	−0.173	0.06
Creatinine#	0.01	0.90
Hypertension	0.365	<0.001
Body height	0.362	<0.001
Body weight	0.563	<0.001
Body mass index	0.456	<0.001

# Value was log transformed for statistical analysis.

Log transformed serum OPG was positively correlated with age (R = 0.409, P<0.001), age equal or more than 60 (R = 0.325, P<0.001) and with an insignificant inverse correlation with BMI (R = −0.158, P = 0.09) and body weight (R = −0.181, P = 0.052). There is no significant correlation between log transformed follistatin, myostatin, OPG, BNP-32 and hsCRP.

Among all the BMD analysis, trochanteric BMD was inversely correlated with age (R = −0.351, P<0.001) as well as femoral neck BMD versus age (R = −0.458, P<0.001) and Ward Triangle region of the hip BMD versus age (R = −0.480, P<0.001). But there is no significant correlation between lumbar region BMD and age (R = −0.109, P = 0.24). Trochanteric BMD in patients with HF was also significantly correlated with BMI (R = 0.456, P<0.001), and also in femoral neck BMD versus BMI (R = 0.499, P<0.001), Ward triangle region of the hip BMD versus BMI (R = 0.332, P<0.001) and Lumbar BMD versus BMI (R = 0.251, P = 0.007). Multivariate analyses of impact of the OPG and clinical factors for trochanteric bone mineral density was conducted, and the inverse correlation of OPG to trochanteric BMD remained after adjusting male gender, age equal or more than 60, smoking, blood urea nitrogen, creatinine, medication of diuretics and body mass index (table 3).

**Table 3:** Multivariate analyses for impact of the osteoprotegerin and clinical factors for trochanteric bone mineral density.

	Model 1	Model 2
	β coefficients	β coefficients
OPG#	−0.233‡	−0.202‡
Male gender	0.323‡	0.291†
Age equal or more than 60	−0.142	−0.080
Hypertension	0.181	0.042
Smoking	0.65	0.035
Creatinine#		0.134
Blood urea nitrogen#		−0.113
Diuretics		0.050
Body mass index		0.365*

OPG: osteoprotegerin.

* p<0.001,

† p<0. 01,

‡ p<0.05.

# data was log transformed for statistic analysis.

Model 1, multivariate analysis after adjusting for male gender, age > = 60, hypertension and smoking.

Model 2, multivariate analysis included in model 2 as well as adjusting for medication use of diuretics and body mass index.

## Discussion

In our study with chronic systolic HF, we found that the worse the HF function classes, the lower the trochanteric BMD and the higher the circulating OPG. In addition, OPG was inversely correlated to the trochanteric BMD in HF patients.

OPG is secreted by osteoblast, mesenchymal stem cells, fibroblasts, endothelial cells and human fat tissue [14]. It is one of the members of the tumor necrosis factor (TNF) receptor super-family and has pleiotropic effects over bone metabolism as well as endocrine function. By binding the receptor activator of nuclear factor- kappa B ligand (RANKL) and acting as a decoy receptor to competitively inhibit RANKL interaction with its receptor activator of nuclear factor- kappa B (RANK), OPG subsequently inhibits the production and differentiation of osteoclasts, as a result, bone resorption is inhibited [15].

HF had been demonstrated with accelerated bone loss and therefore osteoporosis and increased risk of hip fracture [1], [2], possibly attributed to either physical inactivity, increased loop diuretics use [7], similar comorbidities including renal disease, diabetes and altered neuroendocrine such as 25 hydroxyvitamin D [4], IL-1, IL-6 and TNF-alpha [3], [16]. In our study, we had demonstrated an elevated OPG in chronic HF patients which was similar to the other clinical research [9]. In addition, we also found that OPG was inversely correlated to the trochanteric BMD in HF patients.

The reason for having higher circulating OPG level in HF osteoporotic patients may be attributed to several possible mechanisms. First, there may be regulatory pathways that involve bone metabolism other than the RANK-RANKL pathway. There are shared multiple pathophysiological mechanisms for heart failure and osteoporosis, through HF neuroendocrine activation matrix protein abnormalities, parathyroid hormone, adiponectin and leptin, etc [8], [9]. These factors may entangle with OPG in the state of HF. Second, the cause of high OPG in HF may be deduced through similar observation in natriuretic peptides in HF. While natriuretic peptides paralleled HF activity and was utilized in diagnosis, prognosis and monitoring treatment response, natriuretic peptides counteract the detrimental effects of the sympathetic nervous system through diuresis, vasodilatation, inhibition of renin and aldosterone production [17]. The cumulative effects of natriuretic peptides oppose the physiologic abnormalities of HF, instead of begetting HF.

However the relationship of OPG and BMD in distinct disease state is quite controversial from clinical observation. In patients with chronic obstructive lung disease (COPD), a positive correlation was observed between serum OPG and BMD in both lumbar spine L2–L4 and femoral neck region [18]. Adipose tissue OPG expressions are also positively correlated with femoral T score in patient with COPD [19]. On the other hand, in postmenopausal osteoporotic woman, the plasma RANKL, OPG, OPG/RANKL ratio were significant higher than sex-matched control group and circulating levels of OPG and RANKL are inversely related to BMD [20].

In consideration of increased fracture rate in patient with HF, one might expect to observe an osteoporotic range BMD result from HF patient. However, neither our clinical investigation nor other clinical report had yet demonstrated the inference [9]. This may be attributed to the fact that we had precludes initially immobilized, bed ridden, history of joint prosthesis, unable ambulation and renal insufficiency candidates which are the factors that had been associated with osteoporosis. In addition, we had enrolled less severe HF group. For end-stage HF awaiting cardiac transplantation patient, osteopenia and osteoporosis were observed approximately 42% and 19% respectively over femoral neck region [4]. In one study with serial bone mineral density scans over 2 years, accelerated bone loss which defined by a decline over 1% of bone mineral content annually was observed in 35% of HF male patients [21]. The disturbances in bone metabolism and the degree of bone loss may be only subtle in mild to moderate HF patients [22]. Diverse HF medical prescription, including angiotensin-converting enzyme inhibitors, thiazide diuretics, spironolactone and β-blockers with pros and cons effect over osteoporosis may also influence the BMD results in HF patients.

Lumbar spine BMD in our study was relative normal reflecting from Z score in the contrast to hip BMD. This is one interesting but not unique finding, in one study where cardiac transplant recipients and patients with HF awaiting transplantation, hip BMD were decreased while the spine BMD remained normal [23]. HF is associated with several factors that contribute to both reduced bone mineral density and increased risk of osteoporosis-related fractures. Therefore, in theory the systemic impact should bring equal risks to both axial bones and long bones. However, the risk of axial bone osteoporosis and vertebral compression fracture in heart failure was less addressed from the literature. It is postulated that the compressed osteopenic vertebrae bone led to falsely high vertebrae BMD. The discrepancy between femoral neck and lumbar spine BMD may be also caused by aortic calcifications, which may elevate lumbar spine BMD during measurement. In fact, vertebral compression fracture may be often overlooked in contrast to the hip fracture [24].

B-type natriuretic peptide (BNP) and myostatin are both are significantly increased in HF patients. However, there was no significant difference in myostatin among NYHA functional classes, as well as other heart failure associated biomarker such as BNP-32, follistatin in our study. Although Gruson et al. had demonstrated that myostatin may be significantly increased in HF patients and that myostatin may correlate with biomarkers related to HF severity [25]. Zamora et al. proposed that there was no relationship between the myostatin or myostatin propeptide level and any parameter of disease severity or prognosis in patients with chronic heart failure [26]. The insignificant change of myostatin in our heart failure patient may be attributed to the confounding effect of follistatin of which may antagonize myostatin during muscle mass regulation [27]. Angiotensin-converting enzyme inhibitors exert beneficial effects in lowering the risk of weight loss in HF patients and thus the body mass regulation had been altered. In a substudy from the SOLVD database, treatment with enalapril in contrast to placebo, was associated with significant lower risk of weight loss greater or equal than 6% [28]. In addition to the anabolic effect of beta-blockers on bone metabolism, beta-blocker had been also shown to reverse excess protein catabolism [29]. As a result, the absence of expected correlation between myostatin and heart failure severity in our study may be contributed by large portion of angiotensin-converting enzyme inhibitors and beta-blocker use.

There were several limitations to our study. First, this is a cross-sectional, observational association study, lack of clinical outcome. The study population was rather small, heterogeneous, with diversities of medication, which might confound the results. There were group differences in different NYHA classes, which imply that, HF as a disease spectrum, not a single disease. We did not enroll non-HF subjects as controls. This did not deter our observation because we adopted Z-scores from the same region with age-, sex-matched population as the historical controls. However, no serological biomarkers could be assessed. Although the inverse correlation between levels of OPG and BMD which may imply a potential link between HF and osteoporosis were found in the study, it is cautious to jump into conclusion for the causal relationship between them. Heart failure and osteoporosis are complex and multifactorial disorders, and we did not evaluate all potential biomarkers involved. Furthermore, the circulating levels may not reflect tissue levels.

OPG is gaining notice for being a prognostic role in cardiovascular disease. Serum OPG is an independent predictor of cardiovascular mortality in patients with stable coronary artery disease [30] and a markers of plaque instability in coronary artery disease [31]. In GISSI-HF trial, OPG may also pose a prognostic function in chronic HF [32]. Although it had been demonstrate the step up elevation of OPG in HF patients according to NYHA functional class [33], which is concordant with our study result. Few had addressed the change of OPG over the bone metabolism in HF patients. Our study further plots out the interaction between OPG and BMD in HF patients, which is mainly an inverse correlation. OPG may be use as an indicator for osteopenia or osteoporosis clinically. Instead of measuring bone mass directly, the assessment of bone turnover which reflect ongoing bone remodeling mat be alternative for identifying at risk HF patients for osteoporosis and taking preventive and therapeutic measures in time. Although it remains as an unanswered question whether heart failure leads to osteoporosis and frailty fractures, or just being an epiphenomenon as a passive participant in a population at risk for both diseases. However, the application or the mechanism underlies OPG and BMD in HF warrant further randomized prospective, outcome study in larger population and bench works.
